# Physician career satisfaction within specialties

**DOI:** 10.1186/1472-6963-9-166

**Published:** 2009-09-16

**Authors:** J Paul Leigh, Daniel J Tancredi, Richard L Kravitz

**Affiliations:** 1 Center for Healthcare Policy and Research and Department of Public Health Sciences, University of California, Davis, CA.; MS1C, UC Davis Medical School, Davis, Ca, 95616-8638, USA; 2 Center for Healthcare Policy and Research and Department of Pediatrics, University of California, Davis, Medical Center, Sacramento, CA, USA; 3 Center for Healthcare Policy and Research and Division of General Medicine, University of California, Davis, Medical Center, Sacramento, CA, USA

## Abstract

**Background:**

Specialty-specific data on career satisfaction may be useful for understanding physician workforce trends and for counseling medical students about career options.

**Methods:**

We analyzed cross-sectional data from 6,590 physicians (response rate, 53%) in Round 4 (2004-2005) of the Community Tracking Study Physician Survey. The dependent variable ranged from +1 to -1 and measured satisfaction and dissatisfaction with career. Forty-two specialties were analyzed with survey-adjusted linear regressions

**Results:**

After adjusting for physician, practice, and community characteristics, the following specialties had significantly higher satisfaction levels than family medicine: pediatric emergency medicine (regression coefficient = 0.349); geriatric medicine (0.323); other pediatric subspecialties (0.270); neonatal/prenatal medicine (0.266); internal medicine and pediatrics (combined practice) (0.250); pediatrics (0.250); dermatology (0.249);and child and adolescent psychiatry (0.203). The following specialties had significantly lower satisfaction levels than family medicine: neurological surgery (-0.707); pulmonary critical care medicine (-0.273); nephrology (-0.206); and obstetrics and gynecology (-0.188). We also found satisfaction was significantly and positively related to income and employment in a medical school but negatively associated with more than 50 work-hours per-week, being a full-owner of the practice, greater reliance on managed care revenue, and uncontrollable lifestyle. We observed no statistically significant gender differences and no differences between African-Americans and whites.

**Conclusion:**

Career satisfaction varied across specialties. A number of stakeholders will likely be interested in these findings including physicians in specialties that rank high and low and students contemplating specialty. Our findings regarding "less satisfied" specialties should elicit concern from residency directors and policy makers since they appear to be in critical areas of medicine.

## Background

The medical literature is replete with generalizations about morale within individual medical specialties. Internists are "unhappy" and "turning some students away from general internal medicine[1]." "Cardiology is perceived as very demanding in terms of work hours[2]." Surgeons are more negatively impacted by "physical exhaustion" and conflicts between professional and personal life[3]. Obstetricians and gynecologists may be "at risk of burnout[4]." Geriatricians "have the highest job satisfaction of any subspecialty[5]." Stress among psychiatrists dissuades "medical students from choosing psychiatry[6]." Yet these broad assessments are seldom made in comparison to other specialties. Apart from our earlier study, we are aware of no other to compare specialties with a nationally representative sample[7]. But this previous study uses data from 1996-1997 and satisfaction within specialties may have changed[8]. According to a widely cited study, physicians today want more control of lifestyle[8].

Satisfaction is important. Physician satisfaction has been found to strongly correlate with patient satisfaction[9] and desirable patient outcomes[10]. A balance in the specialty mix of physicians is necessary to maintain a high quality of medical care for all Americans[11]. Current dissatisfaction may lead to future declines in numbers of physicians within specialties. Dissatisfied physicians may be more likely to unionize[12], to strike[13], to experience medical problems themselves[14] and to exit medicine altogether[15]. Finally, dissatisfaction may increase rates of medical errors, thus jeopardizing patient safety[16].

This study will update our earlier study with more recent data. Comparative data may help medical school faculty and residency directors to provide medical students with appropriate career counseling, enable medical group managers and policy makers to anticipate workforce trends, and provide practicing physicians with interesting information that could potentially influence career and retirement decisions.

## Methods

### Data

Data were obtained from Round 4 (2004-2005) of the Community Tracking Physician Study (CTS)[17,18]. The survey is a representative sample of physicians, not employed by the federal government, who resided in the continental United States and who provided direct patient care at least 20 hours per week. The survey followed a complex design with a nationally representative sample of 60 communities selected with probability proportional to size (based on estimated population size in July, 1992) from strata defined by geographical region, community size and whether the community is metropolitan or non-metropolitan. These sites were selected in Round 1 of the CTS and have been used in Rounds 1-4 for second-stage sampling of physicians in the so-called *site sample*. For reasons of economy, an independent *national supplemental sample *was not drawn in Round 4, as was done in each of the three previous rounds. At the second stage of sampling in Round 4, an unequal probability sample of physicians was drawn from within each of the 60 sites from strata and sampling classes defined by cross-classifying physicians in the American Medical Association and American Osteopathic Association master files. This classification was carried out according to primary care status and the physician's status and disposition relative to the survey frame and selected sample for the previous round (2000-2001) of the CTS Physician Survey[17] As in previous survey rounds, some hospital-based specialists such as radiologists, anesthesiologists, and pathologists as well as all residents and fellows were excluded, while primary care physicians and responders to previous survey rounds were over-sampled[17,18]. The overall response rate was 53% in 2004-2005[18]. Response rates by physician specialty were not available, but CTS administrators maintain that the data are representative[17,18]. The CTS data are publicly available from the Inter-University Consortium for Political and Social Research at the University of Michigan.

The CTS dataset included information on a total of 6,628 physicians in 2004-2005. For our analysis, we required valid (non-missing) answers for the satisfaction question and for all of the control variables used in the multiple regression model, a restriction that slightly reduced our analysis sample to 6,590 physicians.

### Dependent Variables

The dependent variable was created from answers to this question: "Thinking very generally about your satisfaction with your overall career in medicine, would you say that you are currently... very satisfied, somewhat satisfied, somewhat dissatisfied, very dissatisfied, neither satisfied or dissatisfied, don't know, refused." We coded our satisfaction-dissatisfaction variable as follows: equal to one if the physician stated "very satisfied"; equal to zero for "somewhat satisfied" or "neither"; and equal to negative one for either "somewhat dissatisfied" or "very dissatisfied." "Somewhat dissatisfied" was combined with "very dissatisfied" to increase power since only 4% stated they were "very dissatisfied." "Somewhat satisfied" was combined with "neither" to increase power in the middle value, 0, of our satisfaction score (1 to -1) variable. "Don't know" and "refused" responses were excluded. Roughly 42.7% were "very satisfied" (coded = +1), 42.9% were either "somewhat satisfied" or "neither" (0); and 14.4% were "somewhat" or "very" dissatisfied (-1).

### Specialty Variables

Physician specialty codes classified physicians according to the specialty or subspecialty they reported spending the most time weekly. To enhance the integrity of findings, we combined specialty classifications with fewer than 20 respondents into related specialty classifications to achieve a minimum of 20 respondents in each of the resulting 42 specialty classifications in the present analysis. We also created a three-level lifestyle controllability factor, based upon the Dorsey et al[8] study by grouping specialties. "Controllable" specialties included dermatology, emergency medicine, neurology, ophthalmology, otolaryngology, and child, adolescent, and adult psychiatry. "Uncontrollable" specialties included family practice, general practice, internal medicine, internal medicine and pediatrics (combined), obstetrics and gynecology, orthopedic surgery, pediatrics, general surgery, and urology. "Neither controllable nor uncontrollable" included the remaining 26 specialties in our sample. The CTS did not have data on some specialties mentioned by Dorsey et al^8 ^including anesthesiology, pathology, and diagnostic radiology.

### Control Variables

We selected control variables based on literature review and the Eisenberg Model[19,20]. Variables were classified as physician characteristics, community factors, and practice factors, similar to the previous paper[7].

Physician characteristics included age, gender, race, whether board certified, and whether graduated from foreign medical school. Race was available in 2004-2005, not 1996-1997. Community factors included residence in a town or area with less than 200,000 population (roughly 9%) and residence in nine regions of the country (see Table 1). States were grouped within regions that were defined in the previous study[7].

**Table 1:** Descriptive Statistics on Control Variables, Age Through Foreign Medical School Graduate

**Variable/Description**	**Frequency**	**Percent**	**Mean satisfaction score**	**p-values for uni-variable regression model F-test**
**Entire Sample**	**6,590**	100.0	0.28	
age <35	388	5.9	0.37	0.1223 for 6 age groups
age 35 to 44	2026	30.7	0.30	
age 45 to 54	2256	34.2	0.23	
age 55 to 64	1357	20.6	0.31	
age 65 to 74	448	6.8	0.34	
age 75+	115	1.7	0.31	
White non-Hispanic	4850	73.6	0.30	0.0001 for 5 race categories
African-American non-Hispanic	275	4.2	0.13	
Hispanic all races	338	5.1	0.23	
all other, non-Hispanic	858	13.0	0.29	
missing race	269	4.1	0.08	
gender male	4746	72.0	0.29	0.5216 for 2 genders
gender female	1844	28.0	0.27	
New England	531	8.1	0.25	0.0220 for 9 regions
Middle Atlantic	1005	15.3	0.23	
East North Central	1095	16.6	0.30	
West North Central	220	3.3	0.39	
South Atlantic	1400	21.2	0.24	
East South Central	243	3.7	0.43	
West South Central	698	10.6	0.32	
Mountain	414	6.3	0.29	
Pacific	984	14.9	0.29	
rural/town	815	12.4	0.30	0.6307
board certified	6001	91.1	0.29	0.0244
foreign medical school graduate	1343	20.4	0.23	0.1003

Practice factors included: income, practice ownership, current employment in a medical school, weekly work hours, and experience with managed care. The CTS annual income variable was expressed in dollar units up to a top-code of $400,000. In regression models, we included a rescaled version that expressed incomes in $100,000 units for ease of interpretation of regression results (such that the regression coefficient so generated indicates the change in the satisfaction scale for every $100,000 increase in income). We also included in regression models a dummy variable for incomes reported at the top-code ($400,000 or greater). The three-level CTS practice ownership variable was parameterized with two dummy variables--full owner (sole proprietor) and part owner (partner)--using non-owners as the reference category. We created one variable reflecting whether the physician was currently employed by an academic medical center or school, as opposed to working in private practice or working for a private firm. Work hours were grouped into categories of hours-per-week (≤40; 41-50; 51-60; over 60). The physician's experience with managed care was captured by the variable "percent of revenue from managed care," which was measured in 20 percentage-point units.

### Statistical Methods

Survey design effects arising from unequal probability sampling, stratification and clustering were accounted by using weighting and survey data analysis procedures in Version 9.2 of the SAS System[21] of statistical software programs. Descriptive statistics reported the relative frequency and mean satisfaction scores for each level of the categorical variables.

Mean satisfaction scores were compared in uni-variable and multiple linear regressions. for survey data in SAS PROC SURVEYREG. Survey-adjusted Wald F-tests were used to assess the null hypothesis that mean satisfaction levels were homogeneous across specialties as well as across levels of other independent variables. Regression coefficients for specialties and lifestyle controllability are reported with and without adjustment for control variables. Family practice was chosen as the reference category for specialties for two reasons. First, it contained the most incumbents (n = 1341) of any specialty. Second, family practice percentages for our satisfaction score (0.23) was close to the mean for all specialties (0.28).

## Results

Tables 1 and 2 present relative frequencies and mean satisfaction scores for each of the control variables as well as the "lifestyle controllability" variable. Age range 45-54 contained the plurality of physicians (34%) Roughly 74% were white, non-Hispanic; 4% were African-American, non-Hispanic; and 5% were Hispanic, all races. Females were 28%. Roughly 91% of the sample was board certified and 20% were graduates of foreign medical schools. Roughly 30% were sole proprietors and 22% were business partners. The most frequent work hours category was 41-50 hours (28%). The bottom panel for "lifestyle controllability" indicates that mean satisfaction scores were not equal across these three groups: the average satisfaction score for the "controllable" and the "neither controllable nor uncontrollable" groups were roughly 0.33 whereas the "uncontrollable" group mean satisfaction score was only 0.24.

**Table 2:** Descriptive Statistics on Control Variables, Income through Controllable Lifestyle

**Variable/Description**	**Frequency**	**Percent**	**Mean satisfaction score**	**p-values for uni-variable regression model F-test**
<$50k income	481	7.3	0.23	0.2397 for 7 income categories
$50-$99 k income	801	12.2	0.27	
$100-$149 k income	1656	25.1	0.24	
$150-$199 k income	1387	21.0	0.28	
$200-$249 k income	888	13.5	0.32	
$250-$299 k income	523	7.9	0.28	
>$300 k income	854	13.0	0.34	
work hours ≤40	1763	26.8	0.36	<0.0001 for 4 hours categories
41 ≤work hours ≤50	1850	28.1	0.37	
51 ≤work hours ≤60	1692	25.7	0.24	
work hours ≥61	1285	19.5	0.14	
full owner (sole proprietor)	1966	29.8	0.21	0.0052 for 3 owner categories
part owner (partner)	1442	21.9	0.34	
Not an owner	3182	48.3	0.30	
Currently employed by medical school	628	9.5	0.36	0.0720
Percent revenue managed care 0-20%	1917	29.1	0.30	0.2549 for 4 managed care categories
Percent revenue managed care 21-40%	1669	25.3	0.30	
Percent revenue managed care 41-60%	1281	19.4	0.30	
Percent revenue managed care >60%	1723	26.1	0.23	
**Controllable lifestyle**				
Controllable	1231	18.7	0.33	0.0065
Uncontrollable	4084	62.0	0.24	
Neither controllable nor uncontrollable	1275	19.3	0.33	

Table 3 left-side, provides descriptive statistics for the 42 specialties. Specialties with the greatest numbers of incumbents included family practice (1,341), internal medicine (1,005), pediatrics (740), and emergency medicine (408). Table 3, right-side, provides linear regression results on the 42 specialties, and ranks them based upon the population weighted satisfaction score variable. Each specialty was compared to the satisfaction score for family medicine. The top two statistically significant specialties that were positively associated with satisfaction were pediatric emergency medicine and geriatric medicine. The bottom two statistically significant specialties that were negatively associated with satisfaction were pulmonary critical care medicine and neurological surgery.

**Table 3:** Descriptive Statistics and Regression Results for Specialties; Ranked from High to Low Regression Coefficient, Unadjusted for Covariates ^a^

**Obs**	**Specialty**	**Frequency**	**Mean satisfaction score**	**Regression Coefficient**	**Lower 95% Confidence Limit**	**Upper 95% Confidence Limit**
**1**	Pediatric emergency medicine	29	0.64	0.409**	0.150	0.667
**2**	Geriatric medicine	34	0.57	0.339*	0.066	0.612
**3**	Dermatology	101	0.55	0.312***	0.144	0.480
**4**	Pediatrics	740	0.52	0.283***	0.181	0.385
**5**	Internal medicine and pediatrics	50	0.50	0.268**	0.095	0.442
**6**	Other pediatric subspecialty	98	0.50	0.265**	0.073	0.457
**7**	Neonatal and perinatal medicine	67	0.50	0.264*	0.012	0.516
**8**	Allergy and immunology	55	0.50	0.263*	0.061	0.466
**9**	Child and adolescent psychiatry	59	0.46	0.224*	0.053	0.395
**10**	Radiation oncology	42	0.44	0.202	-0.039	0.443
**11**	Cardiovascular diseases	149	0.43	0.198*	0.014	0.381
**12**	Medical oncology	48	0.43	0.195	-0.097	0.487
**13**	Ophthalmology	184	0.41	0.172*	0.012	0.332
**14**	Occupational medicine	53	0.40	0.166	-0.070	0.402
**15**	Hospitalists	37	0.40	0.165	-0.181	0.510
**16**	Physical medicine and rehabilitation	69	0.39	0.155	-0.027	0.338
**17**	Psychiatry	306	0.37	0.137*	0.010	0.265
**18**	Otolaryngology	81	0.35	0.120	-0.083	0.323
**19**	Other medical subspecialty	20	0.34	0.106	-0.252	0.465
**20**	Critical care internal medicine	29	0.33	0.096	-0.201	0.392
**21**	Endocrinology, diabetes and metabolism	38	0.32	0.088	-0.159	0.336
**22**	Urology	92	0.32	0.084	-0.073	0.240
**23**	Gastroenterology	114	0.27	0.037	-0.137	0.212
**24**	Infectious diseases	36	0.27	0.037	-0.174	0.248
**25**	Pulmonary diseases	56	0.27	0.031	-0.223	0.285
**26**	Other surgical subspecialty	33	0.26	0.026	-0.251	0.304
**27**	General practice	92	0.24	0.005	-0.239	0.250
**28**	Family practice	1341	0.23	referent	referent	referent
**29**	Plastic surgery	52	0.22	-0.011	-0.278	0.255
**30**	Rheumatology	34	0.22	-0.014	-0.245	0.216
**31**	Emergency medicine	408	0.21	-0.020	-0.160	0.119
**32**	Orthopedic surgery	171	0.21	-0.021	-0.175	0.133
**33**	General surgery	216	0.19	-0.039	-0.211	0.133
**34**	Internal medicine	1005	0.19	-0.042	-0.153	0.068
**35**	Neurology	92	0.18	-0.053	-0.202	0.095
**36**	Thoracic surgery	21	0.15	-0.080	-0.529	0.370
**36**	Hematology and oncology	23	0.13	-0.107	-0.345	0.130
**38**	Vascular surgery	33	0.10	-0.129	-0.337	0.079
**39**	Nephrology	50	0.07	-0.159	-0.350	0.032
**40**	Obstetrics and gynecology	377	0.06	-0.173*	-0.317	-0.030
**41**	Pulmonary critical care medicine	31	0.01	-0.228*	-0.419	-0.037
**42**	Neurological surgery	24	-0.36	-0.597**	-1.024	-0.170
	Intercept (family practice)			0.233***	0.159	0.309

^a^P-value legend: ***=<0.001; **=(0.001-0.01); *=(0.01-0.05); F-statistic with 41 and 1602 degrees of freedom = 5.65, p < 0.0001.

A second linear regression was run on the specialties together with the covariates listed in Tables 1 and 2. We present the results from that regression in two tables. Table 4 contains results on control variables and Table 5 on specialties.

**Table 4:** First Set of Regression Results. Control Variables Only (Second Set from Same Regression included in Table 5)^a^

**Covariate**	**Linear Regression Coefficient**	**Lower 95% Confidence Limit**	**Upper 95% Confidence Limit**
age <35	0.146*	0.009	0.283
age 35 to 44	0.100**	0.038	0.161
age 45 to 54	referent	referent	referent
age 55 to 64	0.084*	0.014	0.153
age 65 to 74	0.132*	0.030	0.235
age 75+	0.104	-0.113	0.321
White, non-Hispanic	referent	referent	referent
African-American, non-Hispanic	-0.096	-0.204	0.013
Hispanic	0.011	-0.079	0.102
Asian, Pacific Island, non-Hispanic	0.017	-0.076	0.109
Other race/ethnic	-0.185**	-0.310	-0.060
Male	0.057	-0.012	0.125
New England	-0.024	-0.159	0.110
Middle Atlantic	-0.065	-0.168	0.038
East North Central	-0.001	-0.123	0.122
West North Central	0.082	-0.075	0.239
South Atlantic	-0.058	-0.178	0.061
East South Central	0.143*	0.033	0.252
West South Central	0.017	-0.099	0.133
Mountain	-0.001	-0.142	0.139
Pacific	referent	referent	referent
Rural/town	0.054	-0.054	0.163
Board certified	0.057	-0.019	0.133
Foreign school graduate	-0.053	-0.134	0.028
Continuous income up to $400,000 ($100,000 units)	0.073***	0.035	0.111
Top income $400,000+, binary	0.005	-0.112	0.123
Work hours ≤ 40	0.017	-0.045	0.080
Work hours 41-50	referent	referent	referent
Work hours 51-60	-0.114*	-0.204	-0.024
Work hours >60	-0.201***	-0.280	-0.122
Full owner	-0.076*	-0.141	-0.010
Partner	0.023	-0.052	0.099
Currently employed by medical school	0.105*	0.001	0.209
Percent revenue managed care 20 percent units	-0.020*	-0.038	-0.002

^a^P-value legend: *** = <0.001; ** = (0.001-0.01); * = (0.01-0.05); F-statistic with 71 and 1602 degrees of freedom = 10.61, p < 0.0001

**Table 5:** Second Set of Regression Results. Specialties Only, Ranked by Coefficient (First Set from Same Regression included in Table 4)^a^

**Covariate**	**Linear Regression Coefficient**	**Lower 95% Confidence Limit**	**Upper 95% Confidence Limit**
Pediatric emergency medicine	0.349*	0.042	0.657
Geriatric medicine	0.323*	0.051	0.594
Other pediatric subspecialty	0.270**	0.081	0.459
Neonatal and perinatal medicine	0.266*	0.016	0.515
Internal medicine and pediatrics	0.250**	0.071	0.429
Pediatrics	0.250***	0.146	0.352
Dermatology	0.249**	0.083	0.416
Child and adolescent psychiatry	0.203*	0.019	0.388
Allergy and immunology	0.168	-0.041	0.376
Cardiovascular diseases	0.165	-0.023	0.353
Hospitalists	0.152	-0.224	0.527
Critical care internal medicine	0.144	-0.144	0.433
Psychiatry	0.129	-0.007	0.266
Physical medicine and rehabilitation	0.128	-0.024	0.281
Medical oncology	0.097	-0.201	0.394
Ophthalmology	0.081	-0.080	0.242
Occupational medicine	0.078	-0.162	0.318
Radiation oncology	0.070	-0.183	0.323
Endocrinology, diabetes and metabolism	0.065	-0.234	0.363
Urology	0.064	-0.102	0.229
Pulmonary diseases	0.061	-0.207	0.329
Infectious diseases	0.038	-0.174	0.250
Other medical subspecialty	0.033	-0.305	0.371
Otolaryngology	0.020	-0.180	0.220
General practice	0.012	-0.234	0.258
Family practice	referent	referent	referent
Gastroenterology	-0.002	-0.174	0.170
Internal medicine	-0.005	-0.109	0.100
Other surgical subspecialty	-0.0444	-0.332	0.243
General surgery	-0.053	-0.223	0.116
Neurology	-0.056	-0.200	0.087
Plastic surgery	-0.065	-0.323	0.193
Rheumatology	-0.067	-0.327	0.193
Orthopedic surgery	-0.101	-0.248	0.046
Emergency medicine	-0.130	-0.269	0.010
Thoracic surgery	-0.138	-0.635	0.360
Hematology and oncology	-0.141	-0.377	0.095
Vascular surgery	-0.160	-0.386	0.065
Obstetrics and gynecology	-0.188*	-0.335	-0.041
Nephrology	-0.206*	-0.395	-0.016
Pulmonary critical care medicine	-0.273**	-0.451	-0.094
Neurological surgery	-0.707***	-1.104	-0.309

^a^P-value legend: *** = <0.001; ** = (0.001-0.01); * = (0.01-0.05); F-statistic with 71 and 1602 degrees of freedom = 10.61, p < 0.0001

Considering Table 4, we found a U-shape for age with age<35 and age 65-74 with the highest satisfaction scores. Mean satisfaction scores did not vary significantly with gender. We found only one minor result for race. The category for "no race data" was statistically significant and negatively related to satisfaction. Physicians from the East South Central region reported higher and statistically significant levels of satisfaction than those living in the Pacific region.. Working as a sole proprietor of the practice was negative and statistically significant in its association with satisfaction. A positive association was found for current employment in a medical school. Higher percentages of revenue from managed care were significantly associated with lower levels of satisfaction.

Income and work-hours were the only control variables to generate p-values below 0.001. Income was positively associated with satisfaction. Since we controlled for hours-worked-per-week, this suggested a strong positive association between hourly wage and satisfaction. The two work hour categories for more than 50 hours per-week were strongly negative in their associations with satisfaction.

Table 5 presents ranking of specialties, adjusted for all covariates using linear regression. In comparison to family practice, the highest statistically significant specialties and corresponding regression coefficients were pediatric emergency medicine (regression coefficient = 0.349), geriatric medicine (0.323), other pediatric subspecialties (0.270), neonatal/prenatal medicine (0.266), internal medicine and pediatrics (combined practice) (0.250), pediatrics (0.250), dermatology (0.249), and child and adolescent psychiatry (0.203). The following specialties were significantly more likely than family medicine to be dissatisfied: neurological surgery (-0.707), pulmonary critical care medicine (-0.273), nephrology (-0.206), and obstetrics and gynecology (-0.188).

Table 6 presents linear regression results for the three lifestyle groups, controlling for all covariates in Tables 1 and 2. Because the results on control variables in this Table 6 were so similar to those in Table 4 we omit results on control variables in Table 4. Whereas there do not appear to be statistically significant differences between the "controllable "and "uncontrollable" groups, the "uncontrollable" group was statistically significant with a negative coefficient, indicating the "uncontrollable " group were less satisfied than the referent category, "neither controllable nor uncontrollable."

**Table 6:** Regression Results for Lifestyle Groups, Adjusted for Covariates in Tables 1 and 2 ^a, b^

**Rank**	**Group**	**Regression coefficient**	**Lower 95% Confidence Limit**	**Upper 95% Confidence Limit**
**1**	Neither	referent	referent	referent
**2**	Controllable lifestyle	-0.041	-0.120	0.038
**3**	Uncontrollable lifestyle	-0.072*	-0.135	-0.009

^a^P-value Legend: *** = <0.001; ** = (0.001-0.01); * = (0.01-0.05); F-statistic with 32 and 1602 degrees of freedom = 6.96, p < 0.0001

^b ^Additional covariates in the model include age brackets, race categories, male, regions, residence outside city with population >200,000, board certified, income, work hours, sole proprietor and partner, employed by medical school, and percent revenue from managed care

Table 7 summarizes the statistically significant results on specialties for 2004-05 and compares them with our earlier analysis of 1996-97 data. Since the 1996-97 study ranked specialties separately by "satisfaction" and "dissatisfaction" we re-interpreted our 2004-2005 results as follows: if the regression coefficient was positive and statistically significant, we labeled this specialty as in the "very satisfied " groups and if the coefficient was negative and significant, we labeled this specialty as "dissatisfied." For "very satisfied", there was remarkable consistency from 1996-97 to 2004-05 with geriatric medicine, dermatology, neonatal medicine, and pediatrics at the top of both lists. But only obstetrics and gynecology appeared on both lists for "dissatisfied."

**Table 7:** Summary of Statistically Significant Results from Table 5 and Prior 1996-1997 Study

**Panel A: High values for satisfaction score in 2004-2005 and high percentages for "very satisfied" in 1996-1997**	
**2004-2005**	**1996-1997**
Pediatric emergency medicine	Geriatric internal medicine
Geriatric medicine	Neonatal medicine
Other pediatric subspecialties	Dermatology
Neonatal/prenatal medicine	Pediatrics
Internal medicine and pediatrics (combined practice)	All other specialties (n<40)
Pediatrics	
Dermatology	
Child and adolescent psychiatry	

**Panel B: Low values for satisfaction score in 2004-2005 and high percentages for "dissatisfied" in 1996-1997**.	

**2004-2005**	**1996-1997**
Neurological surgery	Otolaryngology
Pulmonary critical care medicine	Obstetrics and gynecology
Nephrology	Ophthalmology
Obstetrics and gynecology	Orthopedic surgery
	Internal medicine

## Discussion

In this discussion, we first consider the results as well as the literature on specialties and control variables. Second, we consider the implications; third we consider limitations; and fourth, conclusions.

Geriatricians ranked at the top of the statistically significant specialties labeled "very satisfied" in both 2004-2005 and 1996-1997. Shah et al[22] also find high levels of job satisfaction for geriatricians. In addition to the steady (non-erratic) hours, encounters with inspirational seniors, and enduring relationships this specialty is enjoying increasing demand as baby boomers retire[23]. Geriatricians were also high on the list that did not adjust for any covariates (ranked second in Table 3). But caution should be exercised in interpreting these findings. Evidence indicates that relatively poor Medicare reimbursements have lead to shortages of geriatricians nationwide[24].

Pediatrics and pediatric sub-specialties rated high on satisfaction both the 2004-05 and 1996-1997 samples. There may be several reasons: 1) children tend to be more joyful than adults; 2) many health problems are easily resolved so that physicians feel effective; 3) adults who select to work with children may themselves be more joyful; 4) pediatricians encounter less "work stress" than other physicians [25-27].

The 2004-2005 findings again demonstrate the high satisfaction levels and low dissatisfaction levels for dermatology found in the 1996-1997 data. High satisfaction levels for dermatology may be explained by 1) "prosperous employment opportunities[28];" 2) opportunities for preserving business through patient self-referral of their own skin problems; 3) compared to other specialties, dermatologists have more stable work hours; 4) outcomes of treatment are frequently direct and obvious to patients thus enhancing patient-physician interactions[28].

A change also occurred for ophthalmology. In 1996-1997, ophthalmology was statistically significant and high on the list for "dissatisfied" both before and after controlling for income and other covariates. In the 2004-2005 data, ophthalmology was statistically significant and *positively *associated with satisfaction prior to controlling for covariates (Table 3) but not statistically significant after controlling for covariates (Table 5). A literature search and discussions with local experts did not reveal any obvious reasons why changes in satisfaction would have occurred between 1996-1997 and 2004-2005

The low career satisfaction for neurological surgery and obstetrics and gynecology specialists may have several causes: irregular hours, medical malpractice lawsuits; loss of autonomy; and secular decline in pay compared to other specialties[4,29,30]. This low career satisfaction might also be explained by the high expectations these physicians had when they entered these "top tier" specialties versus the current realities of practice. When career expectations are not met, when workers feel cheated, evidence of career dissatisfaction is widespread within most jobs, not just medical ones[31]. Interestingly, these results might generalize to other countries. Lambert et al[32] find evidence that younger physicians in England reject surgical specialties and obstetrics and gynecology for reasons relating to "quality of life" and work hours.

Our results on career satisfaction suggest some effect of lifestyle, especially "uncontrollable" lifestyle. (Bottom of Table 2 and entire Table 6). If the CTS had information on three key "controllable" specialties ---anesthesiology, diagnostic radiology, and pathology---- our multiple regression results for the "controllable " specialties may have been stronger. It is worth noting that two of the "uncontrollable" specialties, pediatrics and internal medicine and pediatrics (combined), rank very high on the satisfaction scale in Tables 2 and 3. Nevertheless, overall, our results parallel those observed among medical students, for whom lifestyle controllability outranked income as an influence on career choice[8]..

The lack of statistical significance for age 75+ might be due to the small "n" within that age bracket(1.7% of sample). The positive and statistically significant results on the remaining age categories and relatively large coefficients in the lowest and highest ranges suggested a U-shaped curve, with physicians age<35 and 65-74 enjoying the highest levels of satisfaction. This might be due to the idealism of youth and the fact that most physicians in retirement age who choose not to retire must enjoy what they do.

Our statistically insignificant results on gender reflect the ambiguity in the literature. McMurray et al[33] find women more dissatisfied than men. Keeton et al[29], on the other hand, find among physicians practicing obstetrics and gynecology, females are more satisfied than males.

We found statistically insignificant results for non-Hispanic African-Americans. The social science literature on many other jobs, however, finds African-Americans more dissatisfied with their jobs[34]. It could be that the medical profession may be one of the first to achieve racial parity for career satisfaction.

Work hours variables measuring many hours (>60, 51-60 hours-per-week) were strongly and positively associated with dissatisfaction, similar to the 1996-97 findings. Work hours appeared to have become an even more important determinant of satisfaction in 2004-05 than 1996-97, consistent with the hypothesis that physicians are becoming increasingly concerned with work-life balance[29].

Unlike the 1996-97 results, these 2004-05 results did not indicate that graduation from a foreign medical school was a statistically significant predictor of lower satisfaction. It may be that the shortage of American physicians has resulted in better career opportunities for international medical school graduates[35,36].

Consistent with the 1996-97 results, higher income continued to be strongly and positively associated with satisfaction. Income, in fact, appeared to be among the most consistent of all covariates in both 1996-97 and 2004-05. This is consistent with economics literature suggesting that income is the most important predictor for most jobs[31]. This is also one of the reasons we reported rankings unadjusted for any covariates in Table 3.

The finding that physicians currently employed in medical schools was unexpected. It could be due to the intrinsic rewards of intellectual stimulation, collaborative research, and creative expression associated with academic life[37].

Consistent with the 1996-97 findings, these 2004-2005 findings also indicate a difference for percent of revenue from managed care. Whereas managed care may be having a waning influence on the public, it may continue to exert influence on physicians[38].

### Implications

As indicated in the earlier study,^7 ^these results might be useful to medical students contemplating specialty choice. Presumably, medical students might select a specialty with high rather than low satisfaction, other things equal.. Specialty societies may also have interest in the results since they are concerned about the well-being of current members and the impression new medical students have of their specialty.

A free market for physicians would operate to improve the lowest ranking specialties since employers and payers would be forced to improve working conditions or wages to continue to attract high-quality personnel. But free market forces are weak in the regulated physician market. Medical group directors, HMO managers, insurance and Medicare executives, policy makers, and residency directors, may want to take direct action to improve career satisfaction among specialties that have especially low scores. Given the strong and consistent relations among income and work hours on the one hand and satisfaction, policy suggestions might include raising payments or reducing work hours for certain specialties. More research is needed to elucidate the reasons for low satisfaction within particular specialties in order to develop policy solutions.

These results may have implications for the future mix of specialists There may be fewer medical students entering obstetrics and gynecology or neurological surgery. Given the critical nature of these specialties, there may also be implications for public health.

### Limitations

First, the data are self-reported. However, only the physician knows his or her level of satisfaction. Secondly, even though a subset of the 2004-05 CTS respondents provided data in earlier survey rounds--with roughly 29% of these respondents providing data in all three earlier rounds--we did not perform a longitudinal data analysis on the subset of respondents who participated in earlier rounds, opting instead for a cross-sectional analysis of the full 2004-05 sample that provides the most contemporary and straightforward look at the broadest range of specialties. It is difficult to assign causal relations using cross-sectional data. But many of the results are consistent with causal relations asserted by other researchers. For example, Clark and Oswald[31] assert that high income improves satisfaction and Becker et al[4] assert that changes in the past 20 years have resulted in growing numbers of dissatisfied obstetrics and gynecology physicians. Third, whereas the response rates may differ across specialties, the CTS administrators believe these data are representative of physicians in the nation[17,18].

Another limitation is our use of a single dependent variable that ranged from +1 to -1. This variable was not normally distributed and linear regression might result in predicted values outside the +1 and -1 range. However, the validity of our regression-based inferences using this dependent variable ultimately rests on the approximate normality of our regression coefficients. Although the quality of this approximation can only be assured by the Central Limit Theorem asymptotically, (i.e. for arbitrarily large sample sizes), we have followed standard practices to promote acceptably accurate approximations[39]. In particular, our scoring of the response variable increased the symmetry of its distribution and we purposely restricted the categorical independent variables in our analyses to those with moderately large number of respondents in each category. A separate problem is that our dependent variable measured satisfaction and dissatisfaction along the same scale. It could be that satisfaction and dissatisfaction are different concepts and require different survey questions for measurement. Alternatively, if could be that this scaling masks the importance of independent variables that have strong but offsetting effects on both satisfaction and dissatisfaction

A final limitation involves some specialties with few incumbents. These include pediatric emergency medicine (n = 29), other medical subspecialties (n = 20), thoracic surgery (n = 21), critical care internal medicine (n = 29), hematology and oncology (n = 23), and neurological surgery (n = 24). Caution should be exercised when interpreting results for these specialties.

We are nevertheless confident in the overall results for several reasons. First, the CTS data are reliable, highly regarded, and used in numerous studies[15,17]. Second, a number of specialties, e.g. pediatrics and obstetrics and gynecology, are well-known for their satisfaction and dissatisfaction and our results coincide with these widespread beliefs. Finally, results on many covariates (income, hours, managed care) are consistent with other studies inside and outside medicine[8,31,32,35-37].

## Conclusion

Career satisfaction varies by specialty. It is important for residency directors, policy makers, physicians and medical students to understand these inter-specialty differences as they make personal, professional, and policy choices.

## Competing interests

The authors declare that they have no competing interests.

## Authors' contributions

DT carried out all statistical analyses, contributed ideas to modeling, interpreted findings, prepared draft paragraphs in the Methods section, reviewed and made suggestions for entire manuscript. RLK provided ideas on modeling, related findings to existing literature, interpreted findings, and prepared drafts of paragraphs throughout manuscript. JPL conceived of the study, wrote most of the paper, interpreted findings, contributed ideas to modeling, and provided general supervision. JPL had full access to all of the data in the study and takes responsibility for the integrity of the data and the accuracy of the data analysis.

All authors read and approved the final manuscript.

## Pre-publication history

The pre-publication history for this paper can be accessed here:


